# Ubiquitin-Proteasome-Collagen (CUP) Pathway in Preterm Premature Rupture of Fetal Membranes

**DOI:** 10.3389/fphar.2017.00310

**Published:** 2017-06-02

**Authors:** Xinliang Zhao, Xiaoyan Dong, Xiucui Luo, Jing Pan, Weina Ju, Meijiao Zhang, Peirong Wang, Mei Zhong, Yanhong Yu, W. Ted Brown, Nanbert Zhong

**Affiliations:** ^1^Lianyungang Maternal and Children's HospitalLianyungang, China; ^2^Peking University Center of Medical Genetics, Peking University Health Science CenterBeijing, China; ^3^China Alliance of Translational Medicine for Maternal and Children's HealthBeijing, China; ^4^Shanghai Children's Hospital, Shanghai Jiaotong University School of MedicineShanghai, China; ^5^New York State Institute for Basic Research in Developmental DisabilitiesStaten Island, NY, United States; ^6^China-US Center of Translational Medicine for Maternal and Children's Health, Southern Medical UniversityGuangzhou, China; ^7^Department of Obstetrics and Gynecology, Nanfang Hospital, Southern Medical UniversityGuangzhou, China

**Keywords:** sPTB, lncRNA, SNV, CNV, collagen, ubiquitin enzymes, UPS, CUP pathway

## Abstract

Spontaneous preterm birth (sPTB) occurs before 37 gestational weeks, with preterm premature rupture of the membranes (PPROM) and spontaneous preterm labor (sPTL) as the predominant adverse outcomes. Previously, we identified altered expression of long non-coding RNAs (lncRNAs) and message RNAs (mRNAs) related to the ubiquitin proteasome system (UPS) in human placentas following pregnancy loss and PTB. We therefore hypothesized that similar mechanisms might underlie PPROM. In the current study, nine pairs of ubiquitin-proteasome-collagen (CUP) pathway–related mRNAs and associated lncRNAs were found to be differentially expressed in PPROM and sPTL. Pathway analysis showed that the functions of their protein products were inter-connected by ring finger protein. Twenty variants including five mutations were identified in CUP-related genes in sPTL samples. Copy number variations were found in COL19A1, COL28A1, COL5A1, and UBAP2 of sPTL samples. The results reinforced our previous findings and indicated the association of the CUP pathway with the development of sPTL and PPROM. This association was due not only to the genetic variation, but also to the epigenetic regulatory function of lncRNAs. Furthermore, the findings suggested that the loss of collagen content in PPROM could result from degradation and/or suppressed expression of collagens.

## Introduction

Spontaneous preterm birth (sPTB) mainly consists of spontaneous preterm labor (sPTL) and preterm premature rupture of the membranes (PPROM). It refers to delivery that occurs before 37 gestational weeks (GWs) and is the leading cause of perinatal morbidity and mortality worldwide (Lawn et al., 2005). Etiologically, sPTB has many causes, including intra-amniotic infection, decidua senescence, and breakdown of maternal-fetal tolerance. The recognized risk factors underlying PPROM include physiologic weakening of the fetal membranes associated with apoptosis near term; dissolution of the amniochorionic matrix exacerbated by contraction-induced shearing forces; infection and inflammation resulting from ascending genital tract colonization initiating a cytokine cascade that triggers membrane degradation; protease production and dissolution of the extracellular matrix (ECM); placental abruption with decidua thrombin expression triggering thrombin-thrombin receptor interactions and increasing choriodecidual protease production; and membrane stretching that may increase amniochorionic cytokine and protease release (Charles and Edwards, 1981; Skinner et al., 1981; Lavery et al., 1982; Taylor and Garite, 1984). The degradation of fetal membranes involved in sPTB is mediated through the activation of Toll-like receptors (TLRs) and causes an increase of matrix metalloproteinases (MMPs; Geraghty et al., 2011; Sandig and Bulfone-Paus, 2012). MMP1 and MMP8 are collagenases that have been found to degrade collagen types I–III and are upregulated in the amnion and chorion (Menon and Fortunato, 2004), which leads to collagenolysis and a decrease in the collagen content of fetal membranes (Draper et al., 1995). The increase in collagen solubility contributes to the remodeling of the ECM and further results in cervical softening and fetal membrane activation (Pollock et al., 1991). Collagen provides the major structural support for the fetal membranes, which is formed by the amnion and chorion. In addition, preterm contractions can accelerate the separation of the amnion and chorion, and then reduce membrane tensile strength, whereas cervical dilation can cause exposure of the membranes to vaginal microorganisms and reduce underlying tissue support (Strohl et al., 2010).

Genetic factors associated with PPROM have been reported. A significant association of a single nucleotide polymorphism (SNP) was found at the genes *MMP1* and *MMP8, CARD15, TLR4*, and *SERPINH1* among PPROM cases (Fujimoto et al., 2002; Wang et al., 2004, 2006). More studies have been carried out on sPTL. The gene loci of *ABCB11, BBS5, FSTL5, CSMD3, NTS, KLHL1*, and *NCAM2*, in addition to duplications at the loci of *OR4P4, OR4S2, OR4C6*, and *RASSF7*, have been shown to be associated with sPTL (Biggio et al., 2015).

Although non-coding RNAs (ncRNAs) are defined by the lack of a protein-coding potential, they have been found to play important roles in many biological processes (Mattick, 2009; Lipovich et al., 2010). The long non-coding RNAs (lncRNAs) are a subtype of ncRNAs with transcripts that are more than 200 nucleotides long without obvious protein-coding potential. Occasionally, lncRNAs may be translated to produce short peptides of unknown function (Fatica and Bozzoni, 2014; Ingolia et al., 2014). LncRNAs predominantly localize to the nucleus and have a lower level of expression than protein-coding regions of genes (Djebali et al., 2012). Based on the biological characteristics of transcription loci and their relationship with the associated genes, lncRNAs can be classified as exonic, intronic, or intergenic overlapping transcripts, in either sense or antisense orientation. LncRNAs may modify the expression of genes and be involved in diverse cellular processes including cell differentiation, imprinting control, and immune responses (Wilusz et al., 2009; Archer et al., 2015). The regulatory function of lncRNAs lies in their ability to alter the expression of DNA in a site-specific manner and, at the same time, bind to different proteins, bridging chromosomes, and protein complexes (Rinn and Chang, 2012; Geisler and Coller, 2013). Evidence increasingly supports the linkage of dysfunctions of lncRNAs to many human diseases, including neurodegenerative, psychiatric diseases (Faghihi and Wahlestedt, 2009), cardiovascular disease (Annilo et al., 2009), and immune dysfunction and auto-immunity (Kino et al., 2010). In our previous study, lncRNAs that are differentially expressed in human placentas delivered from PPROM and sPTL were found to be involved in more than 20 functional pathways (Luo et al., 2013). The patterns of differentially expressed lncRNAs and pathways identified from placentas of PPROM and sPTL were similar to those we observed in our study of human miscarriages (Wang et al., 2014) and of a viral-infected mouse model (Pan et al., 2015), suggesting that deregulation and dysfunction of the ubiquitin-proteasome-collagen (CUP) pathway may be one of the pathogenic mechanisms underlying the adverse outcomes of pregnancies, including PPROM.

On the basis of these findings, we hypothesized that the epigenetic regulatory role of lncRNAs in the ubiquitin proteasome system (UPS) and collagen remodeling is that they are involved in the CUP pathway in sPTB, including PPROM (Zhong et al., 2015). To test our hypothesis, we studied the lncRNAs and lncRNA-associated messenger RNAs (mRNAs) and identified gene mutations/variations associated with the CUP pathway.

## Materials and methods

### Ethics statement

The study design was reviewed and approved by the Ethics Committee of Lianyungang Maternal and Children's Hospital, where all the specimens were collected and stored in an existing biobank, which was developed previously as a core service for the China Preterm Clinical Research Consortium. Written informed consent was obtained from the pregnant women who participated in this study. All material and data were previously coded and are anonymous to the authors of this study.

### Samples

The samples used for the current study were human placentas, fetal membranes, and maternal peripheral blood. Placentas used in microarray hybridization have been described elsewhere (Luo et al., 2013). The criteria for selection of placenta samples were that they were from pregnancies with (1) no clinical signs of infection (no fever, no increase of white blood cell counts, no positive finding of amniotic fluid cultures), (2) no clinical intervention with antibiotics, steroids, or tocolytics during pregnancy, and (3) mother between 25 and 35 years of age. The placental samples were divided into two groups: preterm and full-term. The preterm group (≤35 GW) was further subdivided into PPROM and sPTL. PPROM was defined as a pregnancy that had an initial clinical feature of rupture of membrane that triggered premature uterine contraction. sPTL was defined as the initial sign of labor being uterine contraction without rupture of membrane. The full-term group (between 39^+0^ and 40^+6^ GW), was divided into full-term birth (FTB) and premature rupture of membrane (PROM) at term. Ten samples of human placenta from each group (Table 1)—the sPTL (group A), FTB (group B), PPROM (group C), and PROM (group D)—were subjected to a discovery study with an lncRNA expression microarray (Luo et al., 2013). After the discovery study, 20 fetal membranes from each subgroup were subjected to validation with quantitative RT-PCR (qRT-PCR). The sampling process followed our in-house standard operating procedure. Briefly, immediately after delivery, the separated placentas and/or fetal membranes were rinsed with 200 ml saline twice and dried with sterilized paper towels. Placental tissues were collected with a sterilized scalpel that penetrated completely from the fetal membrane to the decidua as a cube (cm^3^) of 1 × 1 × (2–3.5). A separate piece of fetal membrane (2 × 2 cm^2^) was cut from the amniochorionic membrane (ACM) at the edge of the membrane rupture. The samples were then frozen immediately in liquid nitrogen for a minimum of 30 min before being transferred and stored in a −80°C freezer. An independent subset of 160 maternal blood samples was collected from women shortly before delivery by sPTL, and then was used for isolation of total DNAs followed by exome sequencing. An independent group of 99 women with normal FTB was subjected to sequencing analysis as the controls. These specimens had been previously banked in our existing cohort. The type and size of the samples are listed in Table 1. Comparisons were performed inter-group either individually (such as A vs. B) or combined (such as A+B vs. C+D).

**Table 1:** **Sample size used in the current study**.

**Study**	**Discovery**	**Validation**	**Exome sequencing**
Type of sample	Placenta	Fetal membrane	Whole blood
Number of sample			
Group A (sPTL)	10	20	160
Group B (FTB)	10	20	99
Group C (PPROM)	10	20	
Group D (PROM)	10	20	
Subtotal	40	80	259

### Differential expression profiling of lncRNAs and mRNAs

The Arraystar Human LncRNA Array v2.0 (www.arraystar.com) was the technical platform for the discovery study. qRT-PCR was employed for validation, as reported earlier (Luo et al., 2013; Wang et al., 2014; Pan et al., 2015). In the discovery study, fold changes >2 and *p* < 0.05 were set as cut-offs and were considered significant. In the qRT-PCR study, β-actin (ACTB) was used as an internal control, and the expression values of lncRNAs and lncRNA-overlapped mRNAs were normalized to ACTB. For each RNA, the result of expression level was reported as relative expression by setting the expression value in FTB (subgroup B) at “1,” and the expression value in other groups was calculated relative to this control. The data were subjected to one-way analysis of variance (one-way ANOVA) followed by an unpaired, two-tailed *t*-test. Differences were considered statistically significant at *P* < 0.05. In view of the multiple comparisons that were performed, to minimize the likelihood of a type I error, a Bonferroni correction was applied to the significance criterion (Miller, 1991). This correction is a common methodology to adjust for multiple comparisons that divides the significance criterion (usually 0.05) by the number of comparisons to derive a multiple-comparison-adjusted significance criterion. Additionally, use of FDR (False Discovery Rate) was applied to control multiple tests of correlations (Yekutieli and Benjamini, 1999).

### Whole-genome exome sequencing of sPTLs

Exome sequencing was performed with Hiseq 2000 (Illumina, SanDiego, CA, USA), for which the SureSelect Biotinylated Library (Agilent, Palo Alto, CA, USA) was constructed. The general workflow for calling of single nucleotide variations (SNVs), including SNPs, and of insertions/deletions (InDels) followed vendors' recommendations. Bioinformatic analysis with the Burrows-Wheeler Aligner (Li and Durbin, 2010) was used to align individual “clean data,” and the genotype likelihoods were generated with SAM tools (Szklarczyk et al., 2015). Linkage disequilibrium (LD)–based multiple-sample genotype calling was performed using the LD-based Beagle (Hampson et al., 1997) for multiple-sample genotype calling. Bioinformatic analysis of co-expression and function analysis was performed with the computer programs GeneMANIA (Warde-Farley et al., 2010) and STRING (Szklarczyk et al., 2015).

## Results

### Identification of CUP-associated lncRNAs/mRNAs from human placentas

As shown in Table 2A, nine CUP-associated lncRNAs were identified to be differentially expressed in human placentas, with extremely high statistical significance at *P* < 10^−6^. When the AB groups were compared to the CD groups, three lncRNAs—the ENST00000504601, CR602937, and NR_029434—were found to be upregulated, and two—the AX747492 and AK125314—were downregulated in pregnancies without rupture of fetal membranes. When sPTL (A) was compared to PPROM (C) individually, lncRNA ENST00000413033 was downregulated, but uc.173 was upregulated. LncRNA G42992 was downregulated in PPROM when compared to FTB, and ENST00000482477 was upregulated in PROM (D) vs. FTB (B).

**Table 2A:** **CUP-associated differentially expressed lncRNAs identified from human placentas**.

	**Comparisonregulation**	***P* value**	**FC**	**Seqname**	**Gene symbol**	**Relationship**	**Associated_gene_acc**	**Associated_gene_name**	**Associated protein_name**
**Collagen**	A+B vs. C+D_up	4.8E−07	2.5	ENST00000504601	RP11-893F2.4	Natural antisense	NM_000088	COL1A1	Collagen alpha-1(I) chain preproprotein
	A+B vs. C+D_up	6.2E−20	2.6	CR602937		Natural antisense	NM_030582	COL18A1	Collagen alpha-1(XVIII) chain isoform 1
	A vs. C_down	8.2E−07	2.0	ENST00000413033	RP5-1106H14.1	Intronic antisense	NM_133457	COL26A1	Collagen alpha-1(XXVI) chain
**Ubiquitin Enz**.	A+B vs. C+D_up	1.4E−6	4.1	AX747492	lincRNA-HFE2	Intron sense-overlap	NM_006099	PIAS3	E3 SUMO-protein ligase PIAS3
	A vs. C_up	1.0E−10	3.7	uc.173-	uc.173	Natural antisense	NM_003337	UBE2B	Ubiquitin-conjugating enzyme E2 B
	C vs. B_down	4.9E−14	3.2	G42992		Natural antisense	NM_183399	RNF14	E3 Ubiquitin-protein ligase RNF14 isoform 1
**Proteasome**	A+B vs. C+D_up	2.8E−25	2.0	NR_029434	FLJ31306	Intronic antisense	NM_152132	PSMA3	Proteasome subunit alpha type-3 isoform 2
	A+B vs. C+D_down	2.8E−14	2.1	AK125314		Intron sense-overlap	NM_001128592	PSMG4	Proteasome assembly chaperone 4 isoform a
	D vs. B_up	1.9E−07	2.3	ENST00000482477	AC009299.5	Intronic antisense	NM_005805	PSMD14	26S proteasome non-ATPase regulatory subunit 14

*A, sPTL; B, FTB; C, PPROM; D, PROM; FC, fold change; Seqname, name of lncRNA; acc, accession; Enz, enzyme*.

Forty-nine CUP-associated mRNAs were differentially expressed in human placentas (Table 2B), mostly with considerable statistical significance at *P* < 10^−10^. Among these mRNAs, two were the transcripts of collagen, 22 were ubiquitin enzymes, and four were proteases/proteasomes. Collagen-associated mRNAs (COL-mRNAs) were mainly upregulated in [sPTL+PPROM] vs. [FTB+PROM] and PPROM vs. sPTL, indicating that COL-mRNAs were upregulated in PPROM. Eight mRNAs of ubiquitination enzymes (UBE-mRNAs)—the UBAP1, UBAP2, USP16, USP24, UBE2L6, UBE2Q2, UBE2Z, and UBL3—were identified to be upregulated in PPROM vs. sPTL and downregulated in [sPTL+FTB] vs. [PPROM+PROM]. Seven downregulated UBE-mRNAs (UBAC2, UBE2D3, UBE2E3, UXT, USP20, USP27X, and USP50) in PPROM vs. sPTL were also upregulated in [sPTL+FTB] vs. [PPROM+PROM], suggesting that these eight upregulated and seven downregulated UBEs are associated with PROM in PPROM but not in sPTL. Similarly, the proteasomal protease PSMB8 was upregulated in PPROM vs. sPTL but downregulated in [sPTL+FTB] vs. [PPROM+PROM]. PRSS54 was downregulated in PPROM vs. sPTL, but PRSS33 was upregulated in [sPTL+FTB] vs. [PPROM+PROM].

**Table 2B:** **CUP-associated differentially expressed mRNAs identified from human placentas**.

**CUP**	**Comparison _regulation**	***P* value**	**FC**	**Gene accession**	**Gene symbol**	**Unigene**	**Protein accession**	**Protein**
**Collagen**	A+C vs. B+D_up	5.7E–18	2.76	NM_031361	COL4A3BP	Hs.270437	NP_112729	Collagen, type IV, alpha 3 (Goodpasture antigen) binding protein
	A+C vs. B+D_up	4.2E–05	3.81	NM_000494	COL17A1	Hs.117938	NP_000485	Collagen, type XVII, alpha 1
	C vs. A _up	4.3E–17	3.51	NM_031361	COL4A3BP	Hs.270437	NP_112729	Collagen, type IV, alpha 3 (Goodpasture antigen) binding protein
	C vs. A _up	9.6E–09	6.78	NM_000494	COL17A1	Hs.117938	NP_000485	Collagen, type XVII, alpha 1
**Ubiquitin Enz**.	A+B vs. C+D_up	1.2E–19	2.03	NM_001144072	UBAC2	Hs.508545	NP_808882	UBA domain containing 2
	A+B vs. C+D_up	1.0E–07	2.37	NM_181892	UBE2D3	Hs.518773	NP_871622	Ubiquitin-conjugating enzyme E2D 3 (homolog, yeast)
	A+B vs. C+D_up	1.8E–19	2.73	NM_006357	UBE2E3	Hs.470804	NP_872619	Ubiquitin-conjugating enzyme E2E 3 (homolog, yeast)
	A+B vs. C+D_up	7.8E–19	2.68	NM_153477	UXT	Hs.172791	NP_705582	Ubiquitously-expressed transcript
	A+B vs. C+D_up	1.7E–10	2.11	NM_001110303	USP20	Hs.5452	NP_006667	Ubiquitin specific peptidase 20
	A+B vs. C+D_up	1.8E–19	2.46	NM_001145073	USP27X	Hs.143587	NP_001138545	Ubiquitin specific peptidase 27, X-linked
	A+B vs. C+D_up	2.4E–15	2.18	NM_001098536	USP5	Hs.631661	NP_003472	Ubiquitin specific peptidase 5 (isopeptidase T)
	A+B vs. C+D_up	1.5E–18	2.14	NM_203494	USP50	Hs.677758	NP_987090	Ubiquitin specific peptidase 50
	A+B vs. C+D_down	8.3E–18	2.77	NM_016525	UBAP1	Hs.268963	NP_057609	Ubiquitin associated protein 1
	A+B vs. C+D_down	3.5E–19	2.72	NM_018449	UBAP2	Hs.493739	NP_060919	Ubiquitin associated protein 2
	A+B vs. C+D_down	1.2E–17	2.29	NM_001001992	USP16	Hs.99819	NP_006438	Ubiquitin specific peptidase 16
	A+B vs. C+D_down	3.9E–17	2.54	NM_015306	USP24	Hs.477009	NP_056121	Ubiquitin specific peptidase 24
	A+B vs. C+D_down	3.6E–15	2.01	NM_004223	UBE2L6	Hs.425777	NP_937826	Ubiquitin-conjugating enzyme E2L 6
	A+B vs. C+D_down	1.1E–19	2.39	NM_001145335	UBE2Q2	Hs.23033	NP_775740	Ubiquitin-conjugating enzyme E2Q family member 2
	A+B vs. C+D_down	5.4E–17	2.67	NM_173469	UBE2Q2	Hs.23033	NP_775740	Ubiquitin-conjugating enzyme E2Q family member 2
	A+B vs. C+D_down	1.7E–11	2.10	NM_023079	UBE2Z	Hs.514297	NP_075567	Ubiquitin-conjugating enzyme E2Z
	A+B vs. C+D_down	2.5E–17	2.47	NM_007106	UBL3	Hs.145575	NP_009037	Ubiquitin-like 3
	C vs. A _down	2.1E–10	2.07	NM_001144072	UBAC2	Hs.508545	NP_808882	UBA domain containing 2
	C vs. A _down	1.9E–06	2.51	NM_001110303	USP20	Hs.5452	NP_006667	Ubiquitin specific peptidase 20
	C vs. A _down	2.0E–09	2.27	NM_001145073	USP27X	Hs.143587	NP_001138545	Ubiquitin specific peptidase 27, X-linked
	C vs. A _down	1.2E–08	2.09	NM_203494	USP50	Hs.677758	NP_987090	Ubiquitin specific peptidase 50
	C vs. A _down	4.1E–03	2.07	NM_181892	UBE2D3	Hs.518773	NP_871622	Ubiquitin-conjugating enzyme E2D 3 (homolog, yeast)
	C vs. A _down	5.5E–09	2.70	NM_006357	UBE2E3	Hs.470804	NP_872619	Ubiquitin-conjugating enzyme E2E 3 (homolog, yeast)
	C vs. A _down	1.7E–13	2.18	NM_194259	UBE2I	Hs.302903	NP_919237	Ubiquitin-conjugating enzyme E2I (UBC9 homolog, yeast)
	C vs. A _down	5.5E–08	2.15	NM_153477	UXT	Hs.172791	NP_705582	Ubiquitously-expressed transcript
	C vs. A _up	1.3E–11	3.08	NM_016525	UBAP1	Hs.268963	NP_057609	Ubiquitin associated protein 1
	C vs. A _up	8.8E–08	2.29	NM_018449	UBAP2	Hs.493739	NP_060919	Ubiquitin associated protein 2
	C vs. A _up	2.5E–10	2.03	NM_015902	UBR5	Hs.492445	NP_056986	Ubiquitin protein ligase E3 component n-recognin 5
	C vs. A _up	2.7E–07	2.27	NM_175748	UBR7	Hs.728932	NP_786924	Ubiquitin protein ligase E3 component n-recognin 7 (putative)
	C vs. A _up	8.4E–14	2.88	NM_001001992	USP16	Hs.99819	NP_006438	Ubiquitin specific peptidase 16
	C vs. A _up	2.6E–11	2.33	NM_017414	USP18	Hs.38260	NP_059110	Ubiquitin specific peptidase 18
	C vs. A _up	2.4E–08	2.18	NM_015306	USP24	Hs.477009	NP_056121	Ubiquitin specific peptidase 24
	C vs. A _up	4.8E–10	2.45	NM_001145335	UBE2Q2	Hs.23033	NP_775740	Ubiquitin-conjugating enzyme E2Q family member 2
	C vs. A _up	1.4E–11	2.47	NM_173469	UBE2Q2	Hs.23033	NP_775740	Ubiquitin-conjugating enzyme E2Q family member 2
	C vs. A _up	8.1E–08	2.31	NM_023079	UBE2Z	Hs.514297	NP_075567	Ubiquitin-conjugating enzyme E2Z
	C vs. A _up	2.5E–12	2.85	NM_007106	UBL3	Hs.145575	NP_009037	Ubiquitin-like 3
	A vs. B _up	2.4E–05	2.60	NM_199144	UBE2V1	Hs.727525	NP_954595	Ubiquitin-conjugating enzyme E2 variant 1
	A vs. B _down	6.5E–13	2.03	NM_181762	UBE2A	Hs.379466	NP_861442	Ubiquitin-conjugating enzyme E2A (RAD6 homolog)
**Protease**	A+B vs. C+D_up	2.4E–07	2.09	NM_152891	PRSS33	Hs.280658	NP_690851	Protease, serine, 33
	A+B vs. C+D_down	2.6E–20	2.18	NM_148919	PSMB8	Hs.180062	NP_683720	Proteasome (prosome, macropain) subunit, beta type, 8 (large multifunctional peptidase 7)
	C vs. A_down	6.1E–06	2.20	NM_001080492	PRSS54	Hs.411239	NP_001073961	Protease, serine, 54
	C vs. A_up	1.3E–12	2.27	NM_148919	PSMB8	Hs.180062	NP_683720	Proteasome (prosome, macropain) subunit, beta type, 8 (large multifunctional peptidase 7)
	A vs. B_up	4.1E–05	3.23	NM_002804	PSMC3	Hs.250758	NP_002795	Proteasome (prosome, macropain) 26S subunit, ATPase, 3

*A, sPTL; B, FTB; C, PPROM; D, PROM; FC, fold change; Enz, enzyme*.

### Validation of differentially expressed CUP-lncRNAs and CUP-mRNAs

**Figure 1:**
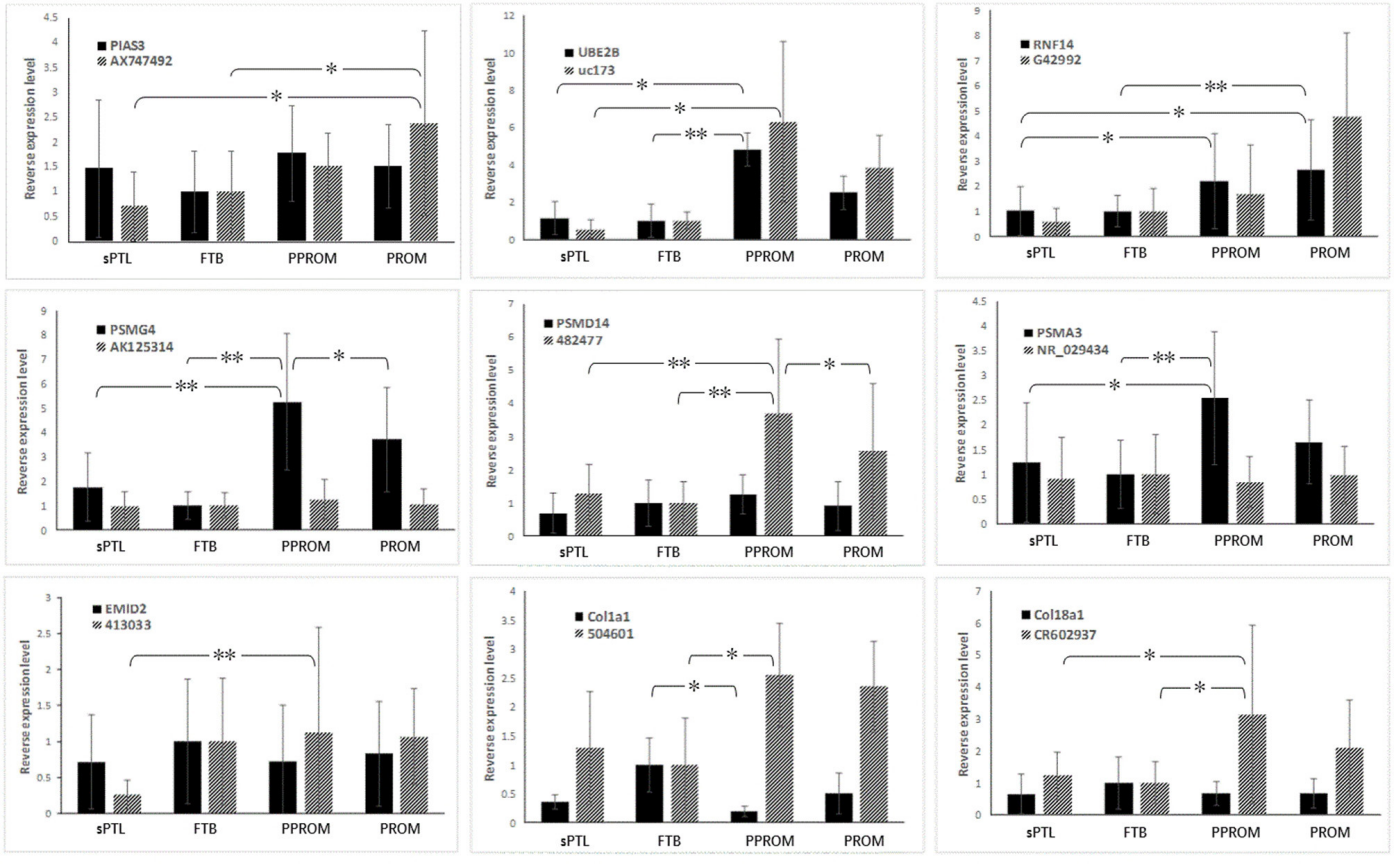
**Expression of CUP-associated lncRNAs and overlapped-mRNAs in human fetal membranes**. Black bars indicate mRNA, and gray bars indicate lncRNAs. ^*^p < 0.05, ^**^p < 0.001.

Nine pairs of lncRNAs and lncRNA-overlapped mRNAs were selected for validation with qRT-PCR. The selection was based on the following criteria: (1) the mRNAs had been found to be differentially expressed between subgroups; (2) the functional product of the mRNAs was involved in either the UPS or collagen remodeling; and (3) the differentially expressed lncRNAs were mostly antisense. The differential expression patterns (DEPs) of these RNAs are shown in Tables 3, 4. In placenta samples, the greatest difference in the expression pattern of RNAs was found between the rupture-of-membrane group [PPROM + PROM] and the labor-without-membrane-rupture group (FTB + sPTL), as nearly all RNAs were transcribed at different levels with statistical significance (*P* < 0.05), except for UBE2B mRNA. When the sPTL subgroup was compared to the PROM subgroup, nine lncRNAs and seven mRNAs were found to be differentially expressed, and when the FTB to PPROM subgroups were compared, eight lncRNAs and seven mRNAs, respectively, were found to be differentially expressed among placentas. When validated with human fetal membranes (the ACMs), however, the DEP of intra-group variations was slightly different from that of placentas (Figure 1).

**Table 3:** **Correlation of differentially expressed CUP-lncRNAs-mRNAs identified from human placentas**.

		**PPROM vs. sPTL**		**PPROM vs. PROM**		**PPROM vs. FTB**		**sPTL vs. FTB**		**sPTL vs. PROM**		**FTBvs. PROM**		**(PPROM+PROM) vs. (FTB+sPTL)**		**(PPROM+sPTL) vs. (FTB+PROM)**	
**lncRNA**	**mRNA**	**lncRNA**	**mRNA**	**lncRNA**	**mRNA**	**lncRNA**	**mRNA**	**lncRNA**	**mRNA**	**lncRNA**	**mRNA**	**lncRNA**	**mRNA**	**lncRNA**	**mRNA**	**lncRNA**	**mRNA**
504601	Col1A1	Up	Up	Up^*^	Down^*^	Down^*^	Up^*^	Down^*^	Up^*^	Up^*^	Down^*^	Up	Down^*^	Down^*^	Up^*^	Up	Down
CR602937	Col18A1	Down^*^	Up^*^	Up^*^	Up^*^	Down^*^	Up^*^	Down^*^	Up	Up^*^	Up	Up^*^	Up^*^	Down^*^	Down^*^	Up	Up
413033	COL26A1	Up^*^	Up^*^	Up^*^	Up	Up^*^	Up	Down	Down	Down^*^	Down^*^	Down^*^	Down	Up^*^	Up^*^	Up	Up^*^
uc173	UBE2B	Down^*^	Up^*^	Up^*^	Up^*^	Down^*^	Up^*^	Down^*^	Down^*^	Up^*^	Down^*^	Up^*^	Up^*^	Down^*^	Down	Up	Up
G42992	RNF14	Down^*^	Down^*^	Up	Up	Down^*^	Down^*^	Down^*^	Down	Up^*^	Up^*^	Up^*^	Up^*^	Down^*^	Up^*^	Up^*^	Down
AX747492	PIAS3	Up^*^	Up	Up^*^	Up	Up^*^	Up	Down^*^	Down^*^	Down^*^	Down^*^	Down^*^	Up	Down^*^	Up^*^	Up	Up^*^
NR_029434	PSMA3	Down^*^	Up^*^	Up	Up	Down	Up^*^	Down	Down^*^	Up^*^	Down^*^	Up^*^	Up^*^	Down^*^	Down^*^	Up	Up
482477	PSMD14	Up^*^	Up^*^	Up	Down^*^	Up^*^	Up^*^	Up	Up^*^	Down^*^	Down^*^	Down^*^	Down^*^	Up^*^	Up^*^	Up	Up
AK125314	PSMG4	Up^*^	Up^*^	Up^*^	Down^*^	Up^*^	Down^*^	Down^*^	Down^*^	Down^*^	Down	Down^*^	Down	Up^*^	Up^*^	Up^*^	Down^*^

* *Statistical significance at P < 0.05*.

**Table 4:** **Verification of CUP-lncRNAs correlated to CUP-mRNAs with fetal amniochorionic membranes**.

		**PPROM vs. sPTL**		**PPROM vs. PROM**		**PPROM vs. FTB**		**sPTL vs. FTB**		**sPTL vs. PROM**		**FTB vs. PROM**		**(PPROM+PROM) vs. (FTB+sPTL)**		**(PPROM+sPTL) vs. (FTB+PROM)**	
**lncRNA**	**mRNA**	**lncRNA**	**mRNA**	**lncRNA**	**mRNA**	**lncRNA**	**mRNA**	**lncRNA**	**mRNA**	**lncRNA**	**mRNA**	**lncRNA**	**mRNA**	**lncRNA**	**mRNA**	**lncRNA**	**mRNA**
504601	Col1A1	Up	Down^*^	Up	Down	Up	Down^*^	Down	Up	Down	Down	Down	Up	Up^*^	Down^*^	Up	Down
CR602937	Col18A1	Up^*^	Down	Up^*^	Down	Up^*^	Down	Up	Down	Down	Down	Down	Up^*^	Up^*^	Down	Up^*^	Down
413033	COL26A1	Up^*^	Up	Up	Down^*^	Up	Down^*^	Down^*^	Down	Down^*^	Down	Down	Up	Up^*^	Down	Down	Down^*^
uc173	UBE2B	Up^*^	Up^*^	Up	Down	Up^*^	Up^*^	Down	Up	Down^*^	Down	Down^*^	Down^*^	Up^*^	Up^*^	Up	Up
G42992	RNF14	Up^*^	Down^*^	Down	Down	Up	Up^*^	Down^*^	Down^*^	Down	Down	Down^*^	Down^*^	Up^*^	Up^*^	Down	Down
AX747492	PIAS3	Up	Up^*^	Down	Up	Up^*^	Up^*^	Down	Down	Down	Down	Down^*^	Down	Up^*^	Up	Down	Up^*^
NR_029434	PSMA3	Down	Up	Down^*^	Up	Down	Up^*^	Down	Up	Down	Down	Up	Down	Down^*^	Up^*^	Down	Up
482477	PSMD14	Up	Up^*^	Up	Up	Up^*^	Up	Up	Down	Down	Down^*^	Down^*^	Up	Up^*^	Up^*^	Up	Up
AK125314	PSMG4	Up	Up^*^	Up	Up	Up	Up^*^	Down	Up	Down	Down	Down	Down^*^	Up	Up^*^	Up	Up^*^

* *Statistical significance at P < 0.05*.

### Co-expression network and functional interactions among CUP-associated genes

**Figure 2:**
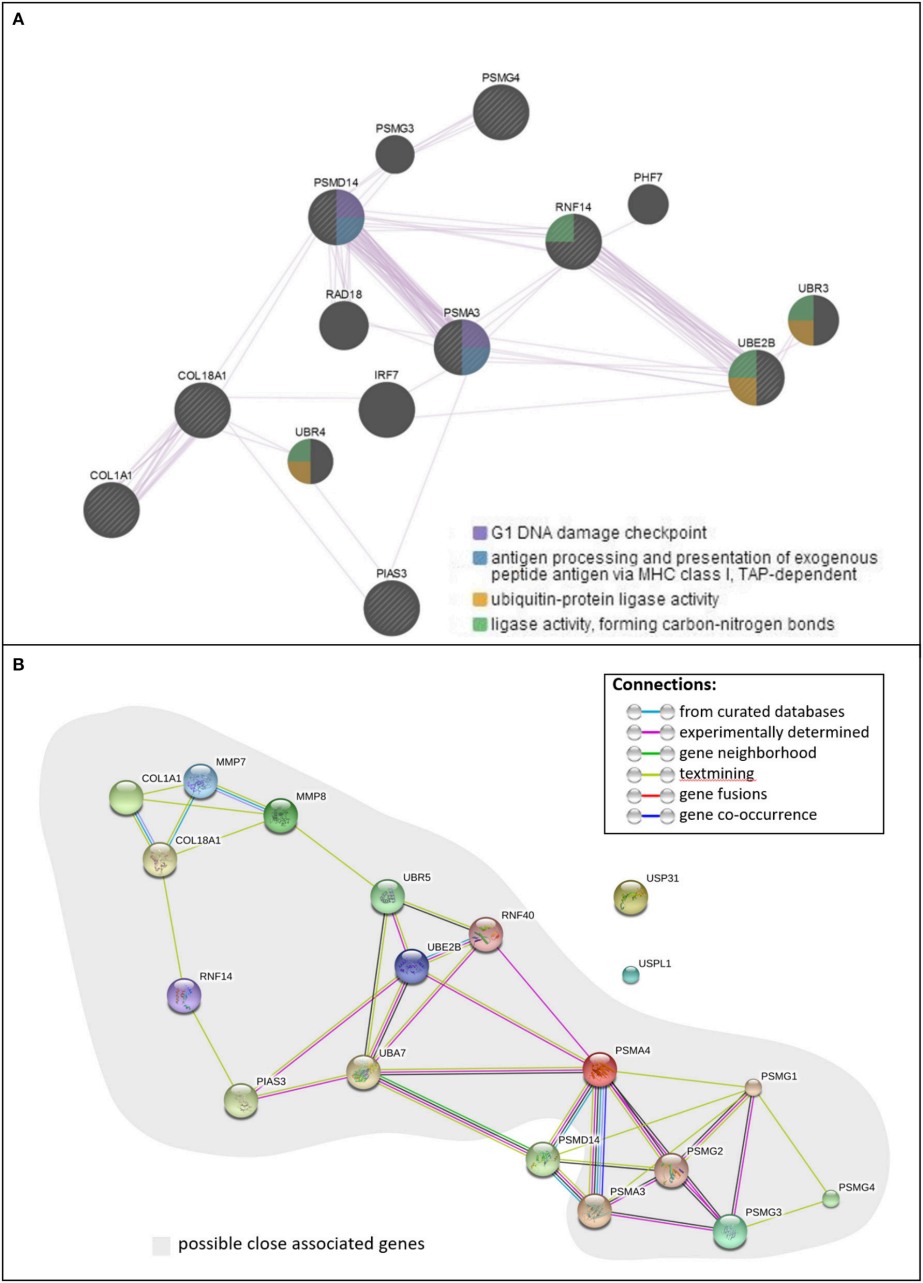
**The functional pathway analysis. (A)** Function and co-expression network of CUP genes, **(B)** interactions of CUP genes.

CUP-associated gene loci, including *COL18A1, COL1A1, EMID2, PIAS3, PSMA3, PSMD14, PSMG4, RNF14*, and *UBE2B*, were subjected to analysis of their network and interactions. As shown in Figure 2A, all eight loci of lncRNA-mRNA pairs were present in the functional network in terms of co-expression. The whole network consists of two intensive co-expressed groups, the collagen group (COL1A1 and COL18A1) and the UPS-related group (PSMD14, PSMG4, PSMA3, UBE2B, RNF14), which were connected by PIAS3 and six other UPS-associated genes. The analysis also showed that PSMD14 and PSMA3 were both involved in the G1 DNA damage checkpoint, antigen procession, and presentation of exogenous peptide antigen via MHC class I. STRING (Szklarczyk et al., 2015) illustrated a similar result (Figure 2B): the proteasome-related genes PSMD14, PSMA3, and five other genes formed shared common protein homology and expression regulation, as did the collagen group, which includes COL18A1 and COL1A1. These two functional groups were then joined by RNF14, PIAS3, and UBE2B through pathways identified in published research articles. Apart from being present in proteasome subunits, PSMD14 and PSMA3 were associated with Epstein-Barr virus infection. PSMD14 consists of JAB1/MPN/MOV34 metalloenzyme domain and was involved in the maintenance of mitochondrial structure and function (Cooper et al., 2009).

### Identification of SNVs including mutations and copy number variations associated with CUP pathway

In the sPTL subgroup, 20 variants, including five mutations, were identified in five collagen genes, three ubiquitin ligase genes, and four ubiquitin-associated proteasome/peptidase genes (Table 5) identified by whole-exome sequencing (Rutishauser et al., 2001). Fourteen of these variations occurred in the intronic region of the gene, and most were deletions. For those exonic mutations, all four were missense, and one was synonymous. Copy number variations (CNVs) were found in four CUP-associated genes from women who delivered by sPTL: COL19A1, COL28A1, COL5A1, and UBAP2. All CNVs were at exons of the genes: three duplications and one deletion (Table 6). The lengths of these CNVs varied greatly, from 700 to more than 388,000 base pairs.

**Table 5:** **Identification of CUP-associated gene mutations/variations from maternal blood specimens from sPTL**.

**Gene**	***P*-value**	**Chrom**	**Position**	**Category**	**Ref_alle**	**Mut_alle**	**Type**	**AA**	**Significance**	**Description**
COL23A1	0.007972	chr5	177987740	exon2	C	T	Point_M	p.G102R	Nonsynonymous	Collagen, type XXIII, alpha 1
COL4A2	0.02807	chr13	111077400	intronic	AT	A	InDel		Del_T	Collagen, type IV, alpha 2
COL4A2	0.04936	chr13	111120699	intronic	C	CT	InDel		Ins_T	Collagen, type IV, alpha 2
COL18A1	0.0086	chr21	46900356	intronic	G	C			SNV	Collagen, type XVIII, alpha 1
COL22A1	0.02772	chr8	139691995	intronic	T	TGTTA	InDel		Ins_GTTA	Collagen, type XXII, alpha 1
COL23A1	0.007972	chr5	177987780	intronic	G	A			SNV	Collagen, type XXIII, alpha 1
COL28A1	0.02708	chr7	7459630	intronic	T	TTATG	InDel		Ins_TATG	Collagen, type XXVIII, alpha 1
COL28A1	0.02755	chr7	7459616	intronic	AT	A	InDel		Del_T	Collagen, type XXVIII, alpha 1
UBE2F-SCLY	0.00987	chr2	239007755	ncRNA_exonic	C	CATT	InDel		Ins_ATT	UBE2F-SCLY readthrough
UBR5	0.002894	chr8	103306033	exon34	T	C	Point_M	p.V1463V	Synonymous	Ubiquitin protein ligase E3 component n-recognin 5
UBR5	0.002894	chr8	103327127	intronic	A	C			SNV	Ubiquitin protein ligase E3 component n-recognin 5
UBA7	0.02729	chr3	49842881	intronic	TG	T	InDel		Del_G	Ubiquitin-like modifier activating enzyme 7
USP31	0.001936	chr16	23102027	exon7	C	A	Point_M	p.D445Y	Nonsynonymous	Ubiquitin specific peptidase
USPL1	0.005818	chr13	31233063	exon9	G	A	Point_M	p.S950N	Nonsynonymous	Ubiquitin specific peptidase like 1
USPL1	0.005818	chr13	31231806	exon9	T	C	Point_M	p.L531S	Nonsynonymous	Ubiquitin specific peptidase like 1
PSMG2	0.02867	chr18	12712817	intronic	C	CATT	InDel		Ins_ATT	Proteasome (prosome, macropain) assembly chaperone 2
PSMA4	0.03335	chr15	78836631	intronic	CTGA	C	InDel		Del_TGA	Proteasome (prosome, macropain) subunit, alpha type, 4

*P val (Rubba et al., 2001), P-value of co-variants; Ref_alle, reference allele; Mut-alle, mutant allele; AA, amino acid; Chrom, chromosome; InDel, Insertion and deletion; Ins, insertion; Del, deletion; SNV, single nucleotide variation*.

**Table 6:** **Identification of CUP-associated CNVs from maternal blood specimens from sPTL**.

**Gene**	**Category**	**Chromosome**	**Starting position**	**Ending position**	**CNV**	**Allele frequency**
COL19A1	exonic	Chr 6	70865930	70866694	Del	0.01
COL28A1, MIOS, RPA3	exonic	Chr 7	7528941	7917273	Dup	0.01
COL5A1, RXRA	exonic	Chr 9	137309042	137623969	Dup	0.01
UBAP2	exonic	Chr 9	33986768	34017213	Dup	0.01

*Chr, chromosome; CNV, copy number variation; Del, deletion; Dup, duplication*.

## Discussion

The current study is a continuing investigation of the regulatory role of lncRNAs in the molecular pathogenesis of sPTB, based on our previous studies, in which we identified differential expression of lncRNAs in sPTL and PPROM (Luo et al., 2013; Pan et al., 2015). In the current study, we analyzed the DEPs of the lncRNAs and the mRNAs that are associated with the CUP pathway that may also associate with PPROM (Zhong et al., 2015). We also identified gene mutations/variations among sPTBs, although a larger sample size will be needed to make a definitive conclusion. These findings provided evidence of the involvement of the CUP pathway in the pathogenesis of PPROM, which could be further supported by the protein interaction network, as shown in Figure 2. Although the algorithms of GeneMANIA (Warde-Farley et al., 2010) and STRING (Szklarczyk et al., 2015) yielded results with slight differences, a clear connection between the collagen and the UPS by an E3 ligase-like protein, PIAS3, was observed (Figure 2B).

Currently, the cause of preterm weakening of fetal membranes leading to PPROM remains unclear. Several studies have concluded the collagen degradation to be the major factor in remodeling of fetal membrane, as the collagen content was lowered in the ruptured membranes (Kanayama et al., 1985; Hampson et al., 1997). The strength of amnion and chorion is basically due to collagen fiber, and the process above reduces the physical strength of the fetal membranes. The major strength in the amnion was shown to be derived from collagen I (extensively in the compact layer and adjacent mesoderm) and collagen IV (a major component of the basement membrane and of the bundles connecting the mesenchymal layer and the epithelium; Bachmaier and Graf, 1999). COL18A1, COL1A1, and EMID2 were all within the collagen family, among which COL1A1 was involved in most human connective tissues, and COL18A1 and EMID2 were shown to be directly associated with the formation and remodeling of the extracellular matrix (Rebhan et al., 1993; Hoffmann et al., 2015). In this study, we have identified both gene mutations and abnormal gene expression. A missense mutation (Table 5) was found within the coding sequence of gene *COL23A1* from sPTL blood samples. The mutation causes the residue of the 102nd amino acid to be changed from glycine to arginine (Wang et al.), which could cause an interruption in the formation of the normal structure of collagen (Lee et al., 1997). It would be interesting to introduce this mutation into the mouse model to investigate whether the missense mutation may generate the sPTL phenotype. Several InDels were identified in the intronic region of collagens from the sPTL cases, indicating that these SNVs may have a genetic predisposition that might function in gene-environmental interactions, in which the environmental factor(s) may induce the epigenetic regulation that consequently may trigger the DEP and influence transcription. In fact, our data showed that mRNAs of COL1A1, COL18A1, and EMID2 (COL26A1) were all downregulated in ruptured membranes in the PPROM subgroup. Interestingly, the expression of lncRNAs 504601 and CR602937, which overlap with COL1A1 and COL18A1, respectively, has been shown to be upregulated in PPROM (Figure 1). Both lncRNAs 504601 and CR602937 are located at the lagging strand as the antisense, opposite from the leading strand of coding genes *COL1A1* and *COL18A1*. Like microRNAs (miRNAs; Jalali et al., 2013), these lncRNAs may also function as a suppressor to down-regulate their complementary mRNAs. Should this hypothesis be confirmed, a novel therapeutic strategy with small interfering RNA could be designed for prevention of sPTB. It would be worth to expand the sPTB cases to replicate our findings in a larger sample size among different ethnic populations globally.

**Figure 3:**
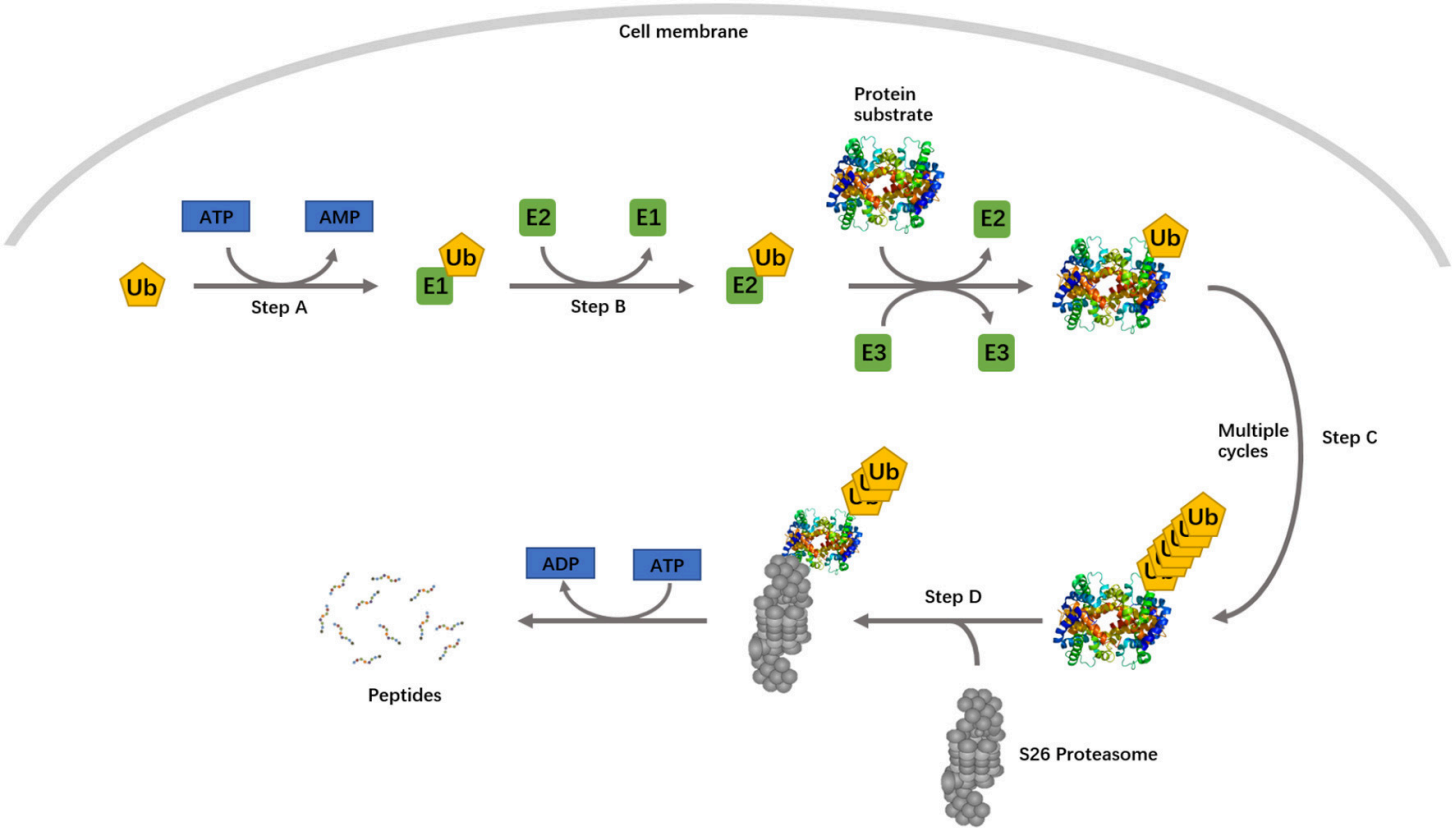
**The ubiquitin proteasome system**. Step A, ubiquitin is activated by a ubiquitin-activating enzyme, E1. Step B, activated ubiquitin is transferred to a ubiquitin-conjugating enzyme, E2. In step C, ubiquitin is subsequently conjugated to target proteins in a process mediated by an E3 ubiquitin ligase. Step D, the polyubiquitylated substrate protein is degraded by the 26S proteasome. A single E1 enzyme can transfer ubiquitin to all E2s in the cells, and each of the E2s associates with a restricted set of E3s that confer substrate specificity.

Differential expression of the lncRNAs and mRNAs of ubiquitin-conjugating protein identified from expression array and qPCR suggested the involvement of the UPS in sPTL and PROM. The UPS is an ATP-dependent, non–lysosomal-proteolytic system. The whole process is shown in Figure 3. The protein product of three genes screened by qPCR belongs to ubiquitin ligase, functioning at different stages of ubiquitination. UBE2B, a member of the ubiquitin-conjugating enzyme family, works as an E2. RNF14 contains a RING zinc finger and can interact with E2s, acting as a ubiquitin-ligase (E3). These genes were found to be overexpressed in PPROM, suggesting that the ubiquitination process was boosted with rupture of the membrane. In the paired lncRNA-mRNA of uc173-UBE2B and G4299-RNF14, there is a clear correlation, in terms of the DEP, between lncRNAs and mRNAs. Apparently, lncRNAs were present as an activator, whereby when the lncRNA is up-regulated in PPROM, the mRNA is up-regulated accordingly. Both lncRNAs uc173 and G4299 are natural antisense. However, they may have a different epigenetic regulatory mechanism, compared to lncRNAs 504601 (COL1A1) and CR602937 (COL18A1). It is likely that the lncRNAs uc173 and G4299 might function as a scaffold to bind to transcriptional factors and facilitate the gene transcription (Engreitz et al., 2016). Previously, Faghihi et al. studied a similar phenomenon in Alzheimer's disease, describing antisense transcripts that can increase mRNA stability by making the binding sites (Faghihi et al., 2008; Faghihi and Wahlestedt, 2009).

The lncRNAs for PSMA3 and PSMD14 were both intronic antisense, but were demonstrated to have a distinct pattern. LncRNA ENST00000482477 presented with the most varied expression pattern, and its level was shown to be greatly increased in PPROM, whereas levels of the lncRNAs AK125314 and NR_029434 remained steady among groups. Like PIAS3, both the PSMG4 mRNA and the lncRNA AK125314 that overlapped with PSMG4 were located at the same (sense) strand, and both were upregulated. Because both lncRNAs and mRNAs are transcribed as the sense strand, the possible mechanism of lncRNAs regulating mRNAs is that lncRNAs may bind to miRNAs, functioning as a sponge, which protects mRNAs from miRNA targeting and repressing. As a result, the transcription of mRNA is upregulated, and the level of mRNA is increased. In this case, the lncRNAs may act as a sink of miRNAs (Poliseno et al., 2010). The paired lncRNA of PSMA3 was intronic antisense. Hawkins and Morris (2010) reported that antisense lncRNAs can bind chromatin and chromatin-modifying proteins, facilitating epigenetic regulation. The expression pattern of PSMD14 and its paired lncRNA ENST00000482477 was similar to that of UBE2B and RNF14; the lncRNAs might regulate their mRNAs by interacting with the miRNA-binding sites.

Whether the differentially expressed lncRNAs that were identified from the human placentas derived from the sPTB are the etiological cause for, or the result of, PPROM is unknown. To better understand this, further investigation of CUP-associated lncRNA expression at the early stage of pregnancy and of the dynamic expression profile of lncRNA longitudinally during the entire pregnancy will be necessary. The lncRNAs in the maternal circulation could be assessed through quantitation of placenta-originated exosomes isolated from maternal blood. Comparing the lncRNA expression profile at early pregnancy and at term-labor may shed light on this important question.

Both pathway analyses indicated the importance of PSMA4 and PSMD14, as they appear to be the central bridge linking the collagens and ubiquitin enzymes to the proteasome proteins. PSMA4 harbors more connections with ubiquitin enzymes (Figure 2). Together with the expression level, we hypothesize that the suppression of collagen and the upregulation of the UPS were functionally connected and then were associated with rupture of the membranes, as a cause or a result, and within the UPS, the proteolysis process was mediated through PSMA4, rather than via PSMD14.

CNVs have been studied extensively for years, and their associations with various diseases have been proved, but the possible association between CNVs and sPTL/PPROM was studied very little in comparison. We applied maternal sera samples in the screening and identified four CNVs affecting seven genes. Results of the study by Biggio et al. of CNVs in more than 1,000 American maternal and neonatal preterm birth samples and term controls showed that only neonatal, not maternal, CNVs were associated with early sPTL (Biggio et al., 2015). However, our previous work on the Chinese population indicated the contribution of maternal CNVs at the RYR1 locus to sPTL (Liu et al., 2013). Research on the Danish population reported the association of maternal CNVs in GSTT1/GSTT2 with smoking, preterm delivery, and low birth weight (Zheng et al., 2013). In the current study, the sample size may not be large enough for statistical evaluations, but the findings nevertheless suggest both race specificity for sPTL and areas for further study on the effects of these involved genes.

In conclusion, differentially expressed lncRNAs and mRNAs, polymorphisms, mutations, and CNVs identified from human placenta and fetal membrane of PPROM supported the involvement of the UPS in the development of PPROM and the possible regulatory pattern of the lncRNAs to their associated mRNAs. The results may also indicate that the loss of collagen content in PPROM was the result of not only degradation, but also of the suppressed expression, of collagen mRNAs. Furthermore, we studied the functional links of collagen to the UPS in PPROM and identified the central connector in proteasome proteins. However, the detailed mechanisms through which lncRNAs regulated their associated mRNAs in sPTL and PPROM must be further studied. On the basis of our data presented here, we propose a “two-hit” hypothesis in which genetic variations/mutations including SNVs and CNVs present as the first hit, which is genetic predisposition. Epigenetic regulation, such as lncRNAs, present as the second hit to modulate the outcome of pregnancy through the lncRNAs' epigenetic regulatory function. Our findings provide a new path for investigating the pathogenesis of sPTL and PPROM.

## Author contributions

The work presented here was carried out in collaboration among all authors. NZ defined the research theme and designed the experiments. XZ, XL, JP, XD, WJ, MZha, and YY were involved in sample collection and carried out the experiments. XZ, XD, PW, WB, and NZ analyzed the data, interpreted the results, and drafted the manuscript. NZ finalized the manuscript. All authors have approved the manuscript.

### Conflict of interest statement

The authors declare that the research was conducted in the absence of any commercial or financial relationships that could be construed as a potential conflict of interest. The reviewer AF and handling Editor declared their shared affiliation, and the handling Editor states that the process nevertheless met the standards of a fair and objective review.
